# Acetyl-Phosphate Is Not a Global Regulatory Bridge between Virulence and Central Metabolism in *Borrelia burgdorferi*

**DOI:** 10.1371/journal.pone.0144472

**Published:** 2015-12-17

**Authors:** Crystal L. Richards, Kevin A. Lawrence, Hua Su, Youyun Yang, X. Frank Yang, Daniel P. Dulebohn, Frank C. Gherardini

**Affiliations:** 1 Laboratory of Zoonotic Pathogens, Rocky Mountain Laboratories, National Institutes of Health, Hamilton, Montana, United States of America; 2 Department of Microbiology and Immunology, Indiana University School of Medicine, Indianapolis, Indiana, United States of America; University of North Dakota School of Medicine and Health Sciences, UNITED STATES

## Abstract

In *B*. *burgdorferi*, the Rrp2-RpoN-RpoS signaling cascade is a distinctive system that coordinates the expression of virulence factors required for successful transition between its arthropod vector and mammalian hosts. Rrp2 (BB0763), an RpoN specific response regulator, is essential to activate this regulatory pathway. Previous investigations have attempted to identify the phosphate donor of Rrp2, including the cognate histidine kinase, Hk2 (BB0764), non-cognate histidine kinases such as Hk1, CheA1, and CheA2, and small molecular weight P-donors such as carbamoyl-phosphate and acetyl-phosphate (AcP). In a report by Xu *et al*., exogenous sodium acetate led to increased expression of RpoS and OspC and it was hypothesized this effect was due to increased levels of AcP via the enzyme AckA (BB0622). Genome analyses identified only one pathway that could generate AcP in *B*. *burgdorferi*: the acetate/mevalonate pathway that synthesizes the lipid, undecaprenyl phosphate (C_55_-P, lipid I), which is essential for cell wall biogenesis. To assess the role of AcP in Rrp2–dependent regulation of RpoS and OspC, we used a unique selection strategy to generate mutants that lacked *ackA* (*bb0622*: acetate to AcP) or *pta* (*bb0589*: AcP to acetyl-CoA). These mutants have an absolute requirement for mevalonate and demonstrate that *ackA* and *pta* are required for cell viability. When the Δ*ackA* or Δ*pta* mutant was exposed to conditions (i.e., increased temperature or cell density) that up-regulate the expression of RpoS and OspC, normal induction of those proteins was observed. In addition, adding 20mM acetate or 20mM benzoate to the growth media of *B*. *burgdorferi* strain B31 Δ*ackA* induced the expression of RpoS and OspC. These data suggest that AcP (generated by AckA) is not directly involved in modulating the Rrp2-RpoN-RpoS regulatory pathway and that exogenous acetate or benzoate are triggering an acid stress response in *B*. *burgdorferi*.

## Introduction

*B*. *burgdorferi*, the agent of Lyme disease in North America, persists in nature in various reservoir animals (e.g., *Peromyscus* species) and is spread to new hosts by a hematophagous arthropod vector (*Ixodes* species) [1, 2]. As the bacterium evolved to a parasitic lifestyle, it sequentially reduced its genome by shedding the genes encoding enzymes used to synthesize essential precursors for RNA, DNA, proteins, lipoproteins and lipids [3]. This effectively locked the bacteria into a host-dependent existence and eliminated the possibility of returning to a “free-living” lifestyle. Successful transmission of *B*. *burgdorferi* between its vector and mammalian hosts requires that the bacteria adapt to changing environmental conditions. Bacteria being acquired by the vector modulate gene expression to promote survival in the tick midgut [4, 5]. During subsequent feeding of ticks colonized by *B*. *burgdorferi*, the bacteria again alter gene expression to promote effective transmission to a new host [4]. Successful transmission of the bacterium to a mammal requires the expression of genes regulated by the Rrp2-RpoN-RpoS regulatory system [6].

First described by Hubner et al., the Rrp2-RpoN-RpoS signaling pathway coordinates the expression of virulence factors that allow *B*. *burgdorferi* to transition between its vector and mammalian hosts [7, 8]. Rrp2 is a response regulator protein that is activated by phosphorylation and associates with the sigma factor RpoN to form a complex that activates RNA polymerase (RNAP) [6, 8]. This Rrp2-RpoN-RNAP complex then activates the expression of the alternate sigma factor RpoS which coordinates expression of key virulence factors that are required for initial infection of the mammalian host [6, 9, 10].

Investigations attempting to identify the phosphate donor for Rrp2 have shown that the cognate histidine kinase (Hk2) and non-cognate Hk1 as well as CheA1, CheA2, and carbamoyl-P were likely not involved [9, 11]. However, the addition of sodium acetate (30, 60, 90 mM) to the growth media resulted in activation of the Rrp2-RpoN-RpoS cascade. It was hypothesized that the Rrp2 activation was due to an increase in the intracellular concentration of acetyl-phosphate (AcP) produced by acetate kinase (encoded by *ackA* or *bb0622*). AckA converts acetate to AcP, which is subsequently converted to acetyl-CoA (AcCoA) by phosphate acetyltransferase (encoded by *pta* or *bb0589*). AcCoA is then condensed to mevalonate for cell wall synthesis [3, 12]. There have been many reports linking AcP to the activation of response regulator proteins [11, 13–16]. However, it is extremely difficult to measure intracellular AcP concentrations in bacteria and Xu et al. were not able to demonstrate that the addition of sodium acetate to the growth medium increased the intracellular concentration of AcP [11, 17].

In this article, we characterize the genes responsible for acetate utilization in *B*. *burgdorferi*. While the complete pathway that converts acetate to undecaprenyl phosphate (C_55_-P) is described here, we have focused on the first enzymes in the pathway, AckA and Pta. Because this is the only pathway that allows *B*. *burgdorferi* to utilize acetate, mutations in *ackA* and *pta* allowed the generation of spirochetes unable to produce AcP or AcCoA. Using these mutants, it was then possible to directly assess the role of AcP in the activation of the Rrp2-RpoN-RpoS signaling pathway. Here we demonstrate that the loss of *ackA* and *pta* had no effect on acetate-, temperature-, or cell density-dependent regulation of the Rrp2-RpoS-RpoN regulatory pathway. Further, analysis of these mutants suggested that the increased activation of the Rrp2-RpoN-RpoS cascade after exposure to acetate is likely due to an acid stress response rather than phosphorylation of Rrp2 by AcP. Based upon the data presented in this study, we conclude that the acetate/mevalonate pathway in *B*. *burgdorferi* is used to generate undecaprenyl phosphate (C_55_-P) and subsequently lipid II, a peptidoglycan precursor, and that the intermediate AcP is not involved in the regulation of the Rrp2-RpoS-RpoN regulatory cascade.

## Results

### Utilization of acetate by *B*. *burgdorferi*

**Fig 1:**
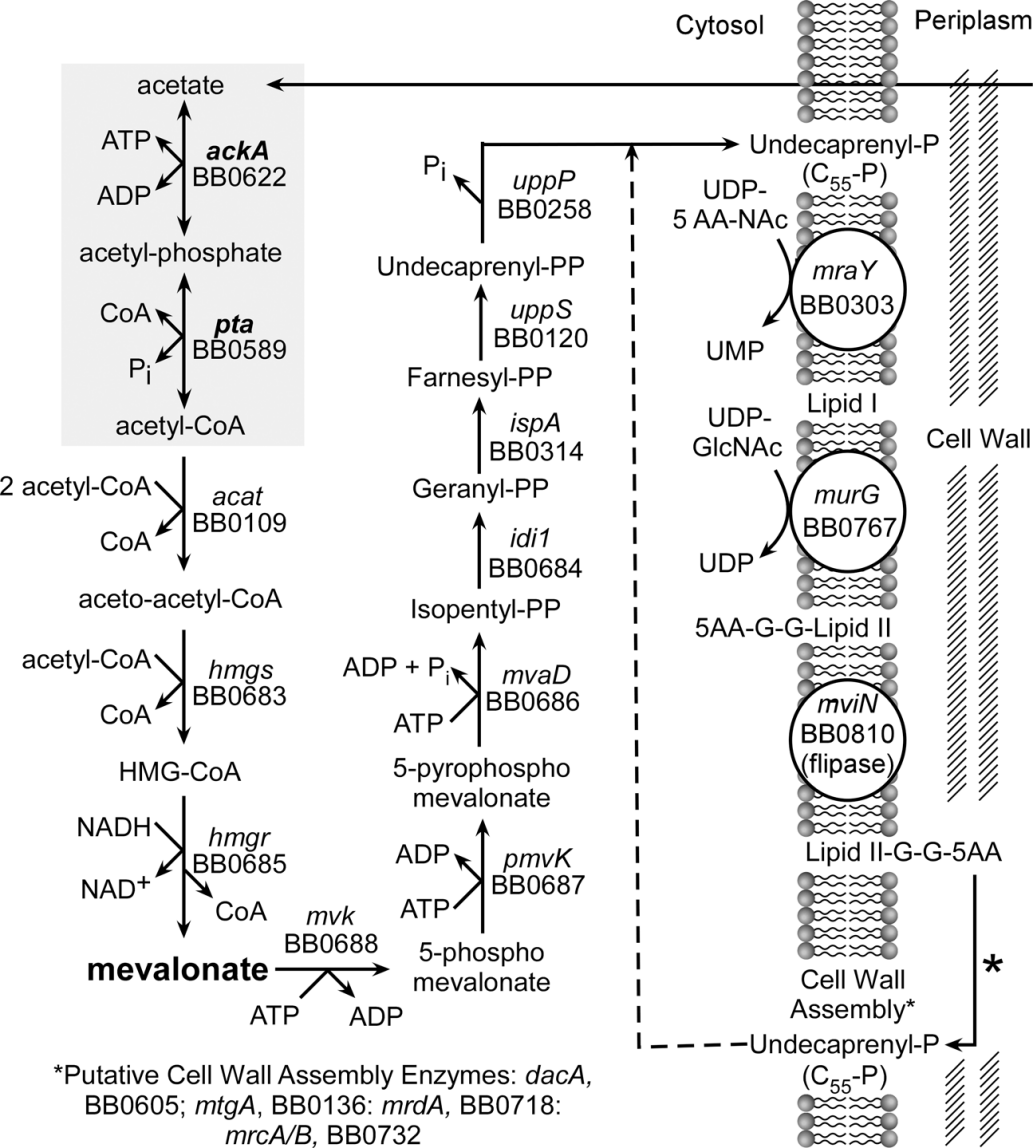
The putative pathway for the synthesis of undecaprenyl-P (C_55_-P) in *B*. *burgdorferi*. A schematic diagram showing the proposed pathway for the conversion of acetate to C_55_-P and its role in cell wall biogenesis. Mevalonate, the intermediate that was added to the plating BSK II to isolate mutants, is highlighted. The genes *ackA* and *pta* are highlighted by shadowing.

Analysis of the genome of *B*. *burgdorferi* identified a complete pathway that encodes enzymes capable of converting acetate to C_55_-P, a peptidoglycan precursor (Fig 1). The genes *bb0622* (*ackA*) and *bb0598* (*pta*) encode enzymes that catalyze the first two steps of this pathway (Fig 1, shaded area). Acetate kinase (AckA) enzymatically catalyzes the conversion of acetate to AcP using a phosphate donated by ATP. Then, phosphate acetyl transferase (Pta) converts AcP to AcCoA which is then condensed to acetoacetyl-CoA and shuttled through the pathway to eventually form lipid II for cell wall biogenesis (Fig 1). In most bacteria, AcCoA and AcP are generated from pyruvate. However, no homolog of pyruvate dehydrogenase has been identified in *B*. *burgdorferi*, suggesting that it is not able to convert pyruvate to AcCoA to generate ATP. In addition, acetate has not been identified as an end product of fermentation in *B*. *burgdorferi* [18]. It has been previously shown that *B*. *burgdorferi* is sensitive to vancomycin, a molecule that binds to and sequesters lipid II (the peptidoglycan precursor ultimately produced by this pathway) [19, 20]. Vancomycin prevents the incorporation of N-acetylglucosamine (GlcNAc)-*N-*acetylmuramic acid (MurNAc)-pentapeptide into newly synthesized peptidoglycan [19, 20]. Sensitivity to vancomycin *in vitro* (MICs: 0.5–2 ug/ml) provides experimental evidence that *B*. *burgdorferi* synthesizes a lipid II moiety structurally similar to other vancomycin sensitive bacteria [20]. These observations and the lack of genes encoding enzymes for fatty acid oxidation strongly suggest that the proposed pathway for the synthesis of lipid II is the only way to generate AcP and AcCoA in *B*. *burgdorferi*.

### Initial characterization of the *ackA* and *pta* loci

**Fig 2:**
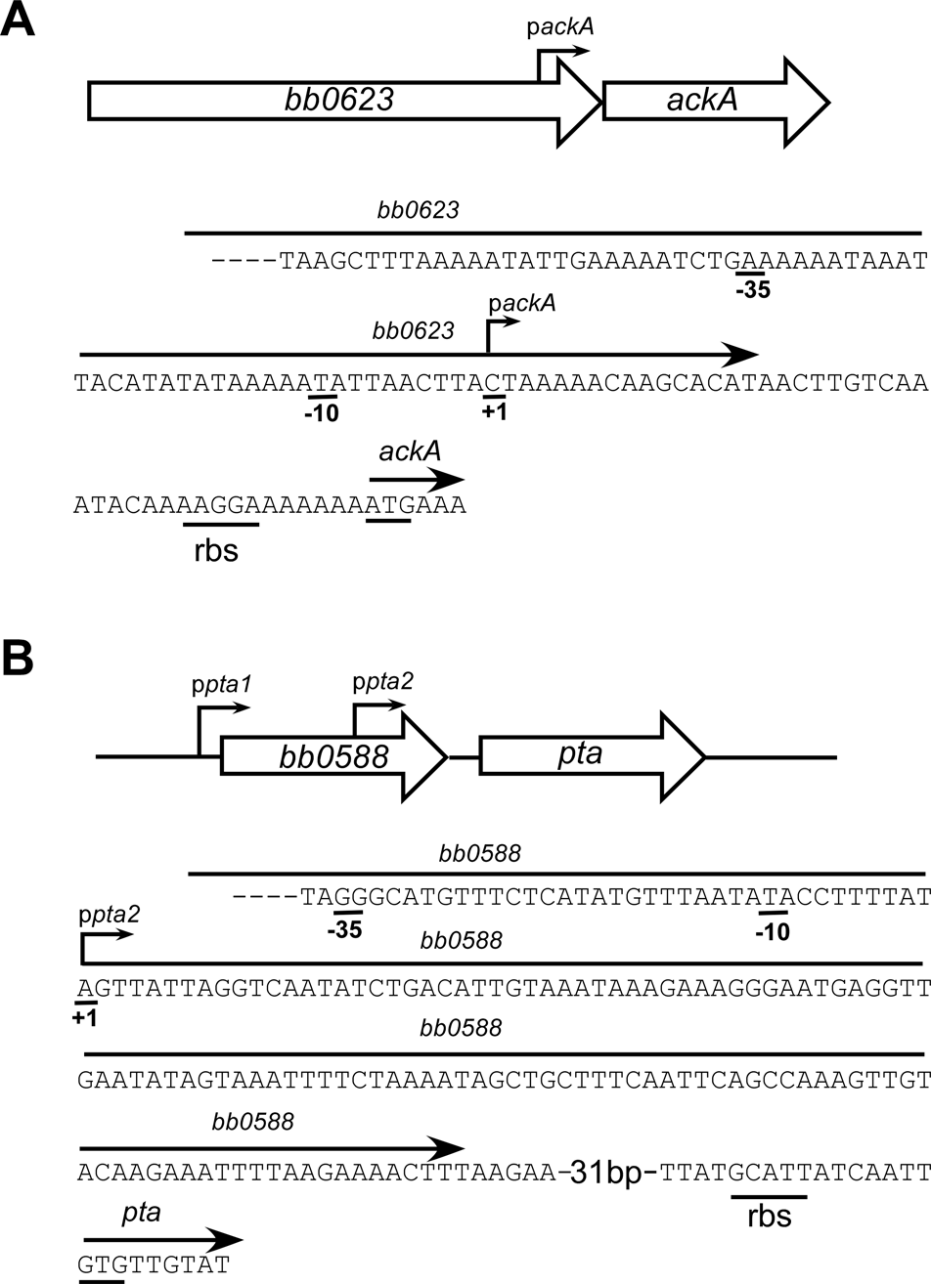
Transcriptional start sites of *ackA* and *pta*. (A) Genomic region and sequence indicating the mapped transcriptional start site of *ackA* (designated p*ackA*). (B) Genomic region and sequence including the mapped transcript start sites of *pta* [designated p*pta1* (reported by Sze and Li, [21]) and p*pta2* from this report]. The putative -35/-10, the ribosome binding site (rbs) and translational start site are underlined for each gene.

It has been proposed that AcP can act as a global signaling molecule in *B*. *burgdorferi* by donating a phosphate to Rrp2 and thereby activating the Rrp2-RpoN-RpoS network of genes [11]. To determine the effect of AcP on gene regulation, *ackA* and *pta* mutants were constructed in wild-type *B*. *burgdorferi* strain B31-A3. In this way, a mutant strain that could not produce AcP or AcCoA by disrupting *ackA*, and a mutant that could over-produce AcP by disrupting *pta*, were generated. Previously, it has been shown that over-expression of Pta had an effect on the expression of genes regulated by the Rrp2-RpoN-RpoS cascade [11]. Since the purpose of this study was to evaluate the role of AcP as a global regulator, it was desirable to complement Δ*ackA* and Δ*pta* using their native promoters to ensure “wild-type” levels of AckA and Pta. Therefore, primer extension analyses were done on RNA isolated from cells harvested in mid-log phase of growth. Analyses showed (four independent biological replicates) that *ackA* had only one transcriptional start site, 42 nucleotides upstream of the predicted start codon (p*ackA*, Fig 2A). Analysis of *pta* was not as clear; multiple assays showed that the transcriptional start for *pta* most commonly mapped 208 bps 5’ to the translational start of *pta* and inside the upstream gene, *bb0588* (p*pta*2, Fig 2B). The promoter reported by Sze and Li (*ppta*1, Fig 2B) is located 5’ to the translational start of *bb0588* but was not identified in any of the analyses performed [21]. In addition, despite multiple attempts, RT-PCR reactions with primers adjacent to the translational start for *pta*, failed to detect transcripts spanning the entire length of *bb0588* and *pta*. Fig 2B depicts the transcriptional start reported here as well as the start reported by [21].

**Fig 3:**
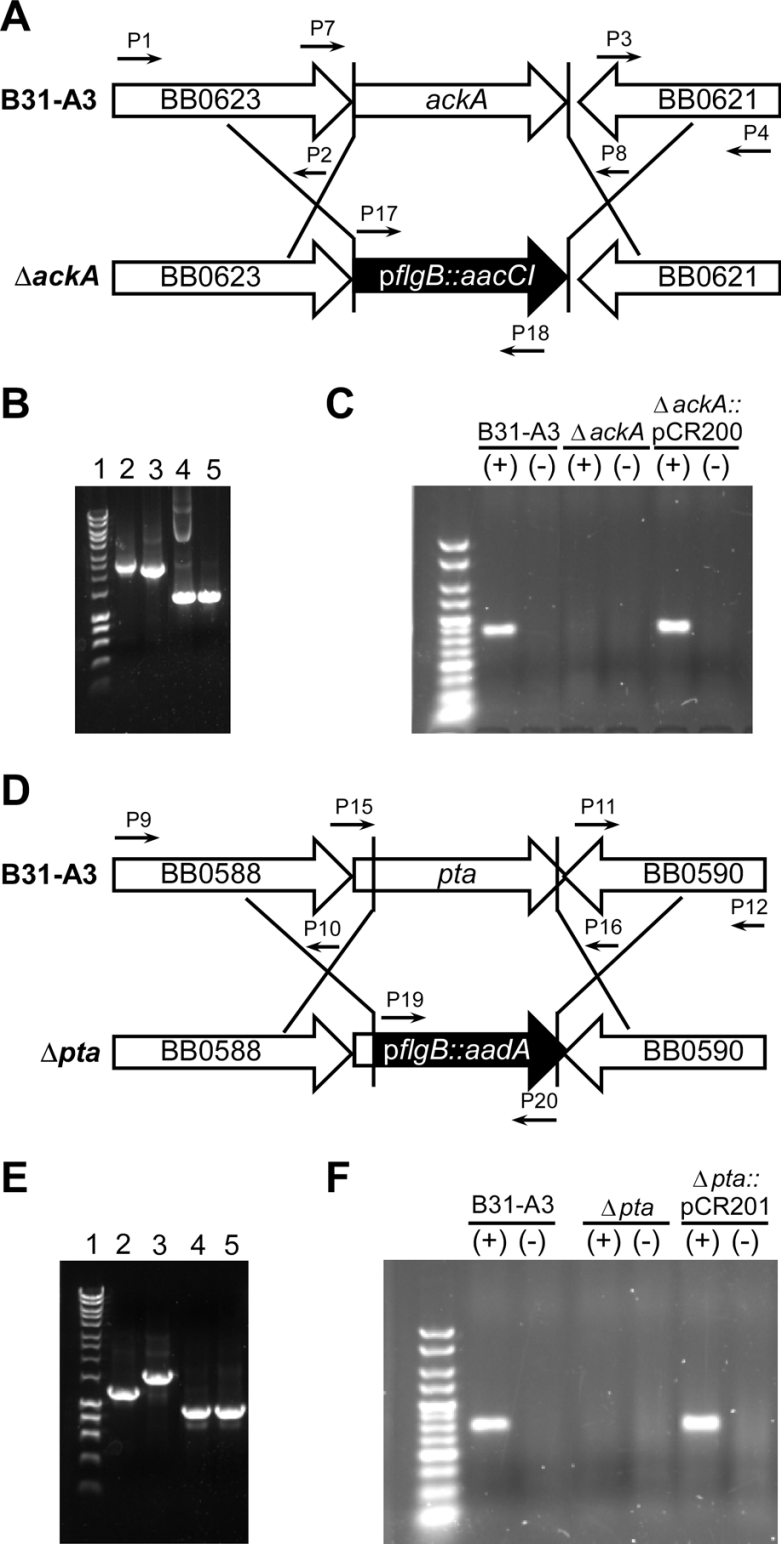
Generating the *ΔackA* and *Δpta* mutants. (A) and (D) Schematic representations of the allelic exchange events that were used to delete *ackA* and *pta*, respectively. (B) PCR of genomic DNA isolated from *B*. *burdorferi* strain B31-A3, Δ*ackA* or Δ*ackA*::pCR200. Lane 1: Hyperladder I, Lanes 2 and 3: the *ackA* locus was amplified from DNA from strain B31-A3 (lane 2) or Δ*ackA* (lane 3) using primers P1 and P4. Lanes 4 and 5: the gentamicin resistance gene, *aacC1*, was amplified from DNA isolated from Δ*ackA* (lane 4) or Δ*ackA*::pCR200 (lane 5) using primers P17 and P18. (C) RT-PCR on DNase treated RNA isolated from the designated strains using primers P21and P22. (+ or -) denotes reactions with or without reverse transcriptase. (E) PCR of genomic DNA isolated from strains B31-A3, Δ*pta* and Δ*pta*::pCR201. Lane 1, Hyperladder I, Lanes 2 and 3, the *pta* locus amplified from genomic DNA from strain B31-A3 (lane 2) or Δ*pta* (lane 3) using primers P15 and P16. Lanes 4 and 5 the streptomycin resistance gene, *aadA*, was amplified using primers P19 and P20 from DNA from Δ*pta* (lane 4) and Δ*pta*::pCR201 (lane 5). (F) RT-PCR on DNase treated RNA isolated from the designated strains using primers P23 and P24. (+ or -) denotes reactions with or without reverse transcriptase.

The deletion of *ackA* and *pta* was accomplished by homologous recombination using allelic exchange vectors that contained the upstream and downstream regions of each target gene flanking the *aacC1* (gentamycin) or *aadA* (streptomycin) resistance cassettes, respectively (Fig 3A and 3D). Mutants were grown in BSKII plating media, supplemented with 10 mM mevalonolactone. This supplement allowed the mutants to survive by bypassing the need for acetate while providing a key intermediate for the synthesis of C_55_-P and cell wall biogenesis (Fig 1). Δ*ackA* and Δ*pta* mutants were isolated and screened for plasmid content and insertion of their respective antibiotic resistance cassettes (Fig 3B and 3E). Δ*ackA* and Δ*pta* mutants isogenic to the parent strain were selected for further characterization. Using data from the primer extension analyses, plasmids pCR200 (*ackA*) and pCR201 (*pta*) were constructed so that both genes would be expressed from their native promoters and both mutant strains (Δ*ackA* and Δ*pta*) were complemented as described in the materials and methods. The genetic makeup of the mutants and their corresponding complements were confirmed by PCR of genomic DNA (Fig 3B and 3E) and by qRT-PCR (Fig 3C and 3F) (Table 1). Strains Δ*ackA*::pCR200 and Δ*pta*::pCR201 were missing plasmid lp25.

**Table 1:** Strains, vectors, and primers used in this study.

**Strains**		**Reference**
*Borrelia burgdorferi* B31-A3 (Low Passage)		[39]
*Borrelia burgdorferi* B31-A3 Δ*ackA*		This study
*Borrelia burgdorferi* B31-A3 Δ*pta*		This study
*Borrelia burgdorferi* B31-A3 Δ*ackA*::pCR200		This study
*Borrelia burgdorferi* B31-A3 Δ*pta*::pCR201		This study
*Escherichia coli* TOP10		Invitrogen
**Plasmids**		**Reference**
pPCR-Script Cam SK(+)		Agilent
pBSV2G		[40]
pKFSS1		[41]
pCR100 (pPCR-Script Cam SK+::Δ*ackA*::aacC1)		This study
pCR101 (pPCR-Script Cam SK+::Δ*pta*::aadA)		This study
pCR200 (pKFSS1::*ackA*)		This study
pCR201 (pBSV2G::*pta*)		This study
**Primer**	**Sequence 5'–3'**	**Reference**
1. ackA1-kpnI	ACTGCTGGTACCAGGTGTTGGCAATTTACTTGG	This study
2. ackA1-xhoI	ACTGCTCTCGAGGTTATGTGCTTGTTTTTAGTAAGT	This study
3. ackA1-claI	ACTGCTATCGATTAATTTACAATTTTAAAAACTAAAATCT	This study
4. ackA1-pstI	ACTGCTCTGCAGTGCAAATGGCTTTGAAGATATTGAGG	This study
5. ackA2-kpnI	ACTGCTGGTACCGTGGATTTATTCCTGAAAATTATG	This study
6. ackA2-sphI	ACTGCTGCATGCTAAATTTTTTGGAATTAAATTATAAATGTC	This study
7. ackA-F	CAGGGCTTTGGAAATAGAATACA	This study
8. ackA-R	GGTCTTCTAGGATTCAATAAGTTTACATGTTATCC	This study
9. pta1-kpnI	ACTGCTGGTACCTTAATTCTGGCGTTGCTGGT	This study
10. pta1-xhoI	ACTGCTCTCGAGCGGGAAAAACTATATTGGCCTTA	This study
11. pta1-claI	ACTGCTATCGATGCCCATTAGCGATCTTTCAA	This study
12. pta1-pstI	ACTGCTCTGCAGAACCGCATTTGAAATTGACTT	This study
13. pta2-xhoI	ACTCTCGAGTTTAATGCCAATAAAAATTTAATTAAGAATGC	This study
14. pta2-pvuI	ACTCGATCGTTAAATGCTTATCATTAAAGC	This study
15. pta-F	GTTGTACAAGAAATTTTAAG	This study
16. pta-R	CATTACCAATAAAGCTACTCTG	This study
17. aacC1-xhoI	ACTGCTCTCGAGTAATACCCGAGCTTCAAGGA	This study
18. aacC1-claI	ACTGCTATCGATTTAGGTGGCGGTACTTGGGTCGAT	This study
19. aadA-xhoI	ACTGCTCTCGAGTACCCGAGCTTCAAGGAA	This study
20. aadA-claI	ACTGCTATCGATTTATTTGCCGACTACCTTG	This study
21. ackA-RTF	TGCAATCACCGCAATAGAAG	This study
22. ackA-RTR	GAAAGGCCATGAAAGCCATA	This study
23. pta-RTF	TGCAGCTTGATTCAGCCATA	This study
24. pta-RTR	CCTTGGCAAAAGCAAATCTC	This study
25. rpoD-RTF	GCAGGCTGAAAATTGAAAAA	This study
26. rpoD-RTR	CAAGTTCTTTTTGGGCAAGC	This study
27. ospC-RTF	TGCGGTTTTACTTGCTGTGA	This study
28. ospC-RTR	ATTGCATAAGCTCCCGCTAA	This study
29. rpoS-RTF	AGGCAATGCAAAAGCAAAAA	This study
30. rpoS-RTR	TCGGGTCATATTTTTCAGCA	This study
31. ackA-GSP1	GCTTGATCCTCCATGTACAAC	This study
32. ackA-GSP2	GCTAAGAGTTTTAAGGATTTT	This study
33. pta-GSP1	CCCTTTAACTTTTGTAAACTC	This study
34. pta-GSP2	CTGGGAAAGAATTAGGAT	This study
35. pta-GSP3	GCAATTAGAAAATTCTTT	This study

The underlined sequence identifies the restriction site within each primer.

### The Δ*ackA* and Δ*pta* mutants require mevalonolactone for growth and viability

**Fig 4:**
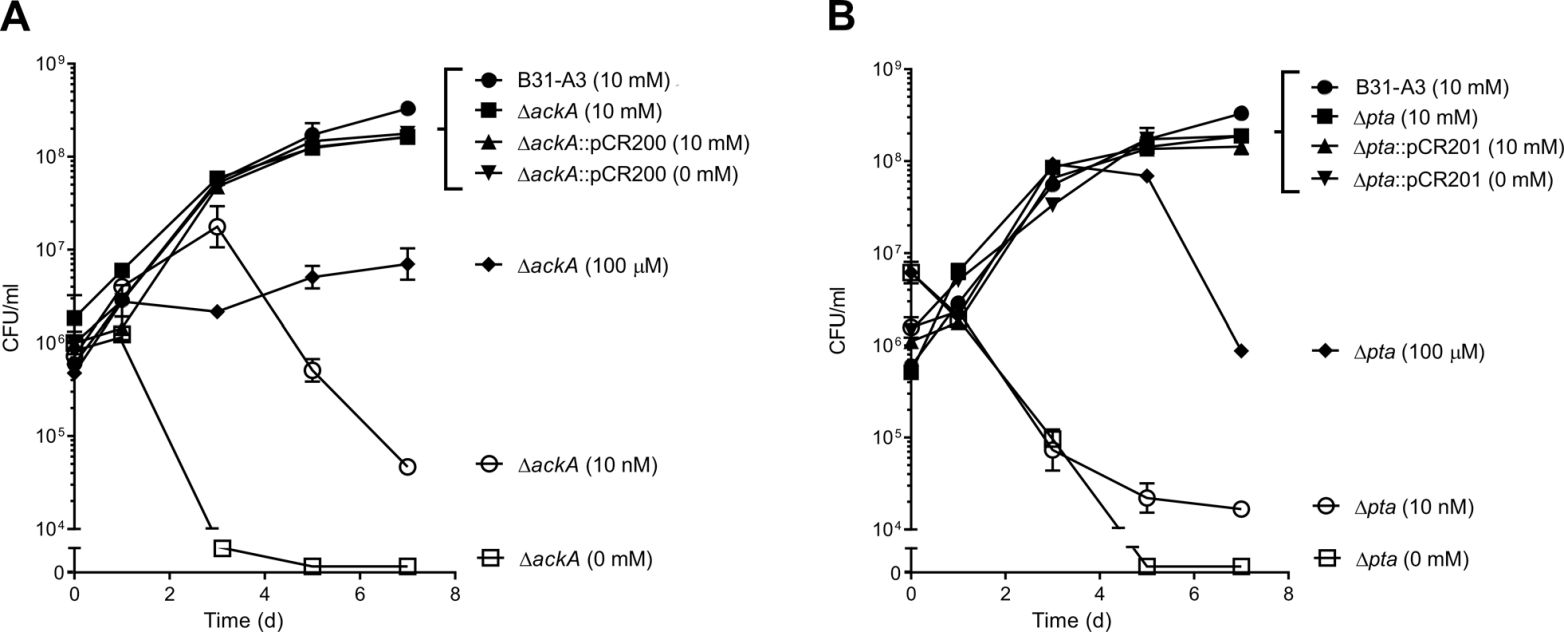
Growth and survival of *B*. *burgdorferi* B31-A3, Δ*ackA*, Δ*pta*, Δ*ackA*::pCR200 and Δ*pta*::pCR201 in the presence and absence of mevalonolactone. Growth of strains B31-A3, Δ*ackA*, and Δ*ack*::pCR200 (A) and B31-A3, Δ*pta*, and Δ*pta*::pCR201 (B) in BSKII supplemented with increasing concentrations of mevalonolactone (0–10 mM, shown in parentheses). Cells were quantified by culture in BSKII plating media supplemented with 10 mM mevalonolactone.

To generate Δ*ackA* and Δ*pta*, the metabolite mevalonolactone was required as a supplement in the BSKII growth medium. Commercially available mevalonolactone undergoes hydrolysis by water and forms an equilibrium between mevalonolactone and mevalonate at neutral pH. After diffusing into the cell, mevalonate is converted to phosphomevalonate by mevalonate kinase (*bb0688*) and is then presumed to generate C_55_-P (Fig 1). Since C_55_-P is essential for growth, strains Δ*ackA* and Δ*pta* should have an absolute requirement for exogenous mevalonate. The requirement for mevalonate was tested in strains B31-A3, Δ*ackA*, Δ*pta*, Δ*ackA*::pCR200 and Δ*pta*::pCR201 (Fig 4). All strains were grown in BSKII supplemented with increasing concentrations of mevalonolactone (10 nM to 10 mM). The minimum concentration of mevalonolactone that could support growth and viability of Δ*ackA* and Δ*pta* was 100 μM; cells exposed to concentrations below that threshold showed decreased viability, eventually leading to cell death (Fig 4). The addition of mevalonolactone did not appear to effect the growth of wild-type *B*. *burgdorferi* and complementation of Δ*ackA* and Δ*pta* resulted in a complete restoration of growth and viability without mevalonolatone in the growth medium (Fig 4).

### Influence of the *ackA* and *pta* mutations on RpoS and OspC expression following a shift in growth temperature

**Fig 5:**
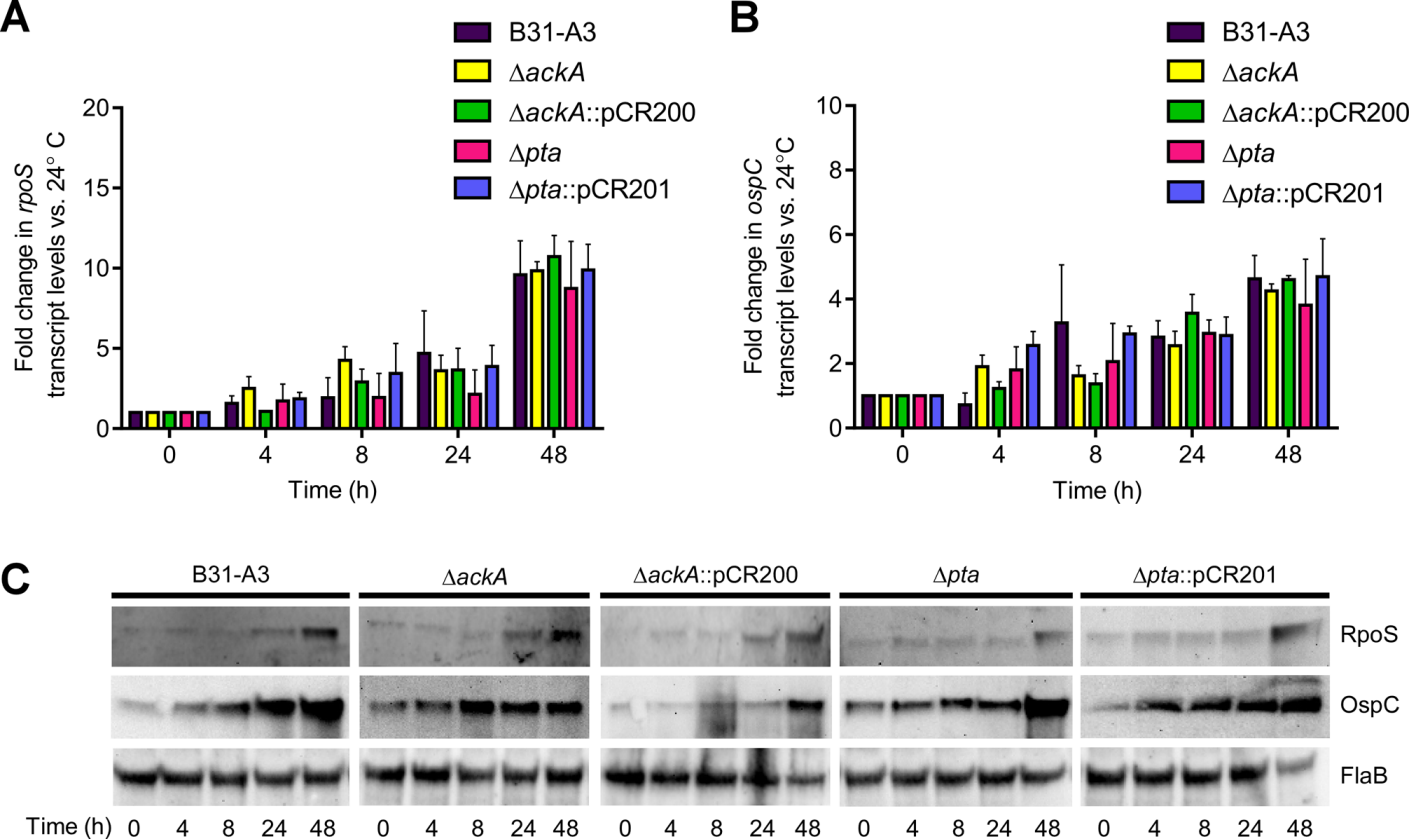
Quantitative RT-PCR of *rpoS* and *ospC* and immunoblot analysis of RpoS and OspC in strains B31-A3, Δ*ackA*, Δ*pta*, Δ*ack*::pCR200 and Δ*pta*::pCR201 after an increase in growth temperature. (A) RT-PCR analysis of *rpoS* and (B) *ospC* transcripts in B31-A3,Δ*ackA*, Δ*ackA*::pCR200, Δ*pta*, and Δ*pta*::pCR201, after a temperature shift from 24°C to 34°C. Samples were collected at 0, 4, 8, 24, and 48h and transcript levels were compared to expression at 24°C. (C) Immunoblot analysis of RpoS and OspC in each strain after a temperature shift and sampling at the indicated time points. Immunoblots were probed with antiserum specific for RpoS, OspC and FlaB. FlaB was included as a protein load control for each assay.

Previous studies have attempted to identify potential phosphodonors in *B*. *burgdorferi* that activate Rrp2 and stimulate the RpoN-RpoS regulatory cascade [9, 11]. B31-A3, Δ*ackA* and Δ*pta*, and their respective complemented strains were assayed for changes in the transcription and translation of *rpoS* and *ospC* under conditions that have been shown to activate the Rrp2-RpoN-RpoS regulatory cascade [4, 5, 22]. One environmental signal/condition known to activate the Rrp2-RpoN-RpoS regulatory cascade is an increase in temperature (24–34°C). After a shift from 24°C to 34°C, gene transcript levels were assayed by qRT-PCR in *B*. *burgdorferi* B31-A3, Δ*ackA*, Δ*pta*, Δ*ackA*::pCR200 and Δ*pta*::pCR201. Cultures were harvested after 4, 8, 24, and 48 hours (prior to cultures reaching stationary phase) in order to distinguish temperature-dependent RpoS activation from cell density-dependent regulation. *rpoS* and *ospC* transcript levels were compared with transcript levels of cells grown at 24°C and normalized to *rpoD* transcripts. As shown in Fig 5A, when compared with cultures maintained at 24°C, there was slight variation in *rpoS* transcript between all five strains tested at the early time points (4 and 8h). At 24 and 48h post shift in growth temperature, all five strains had similar increases of *rpoS* transcription. At 48h post temperature shift, *rpoS* transcription increased dramatically (~7 to 10-fold) as cells reached a cell density of ~7–9 x 10^7^ cells ml^-1^. The observed changes in *ospC* transcript levels followed patterns similar to that observed with *rpoS* transcripts (Fig 5B). However, there was more variability between strains at the 4 and 8h time points when compared to *rpoS* transcript levels. Each strain showed increased *ospC* transcript levels, similar to trends seen in wild-type strains as described previously [9].

Immunoblot analyses were also used to assess RpoS and OspC synthesis in the five *B*. *burgdorferi* strains following an increase in growth temperature. RpoS levels were virtually undetectable at early time points but increased with time as cultures were incubated at 34°C (Fig 5C). OspC could be detected at every time point assayed and increased concomitantly with an increase in *rpoS* and *ospC* expression. Although there was minor variation, there were no dramatic differences in protein profiles of the five strains. Each strain increased the RpoS and OspC levels in response to growth at an increased temperature, consistent with previous studies [9, 22]. Overall, these qRT-PCR and immunoblot analysis suggest that AcP, synthesized by AckA, was not required to activate the Rrp2-RpoN-RpoS cascade following a temperature shift.

### Influence of the *ackA* and *pta* mutations on RpoS and OspC expression when *B*. *burgdorferi* cells reached stationary phase/maximum cell density

**Fig 6:**
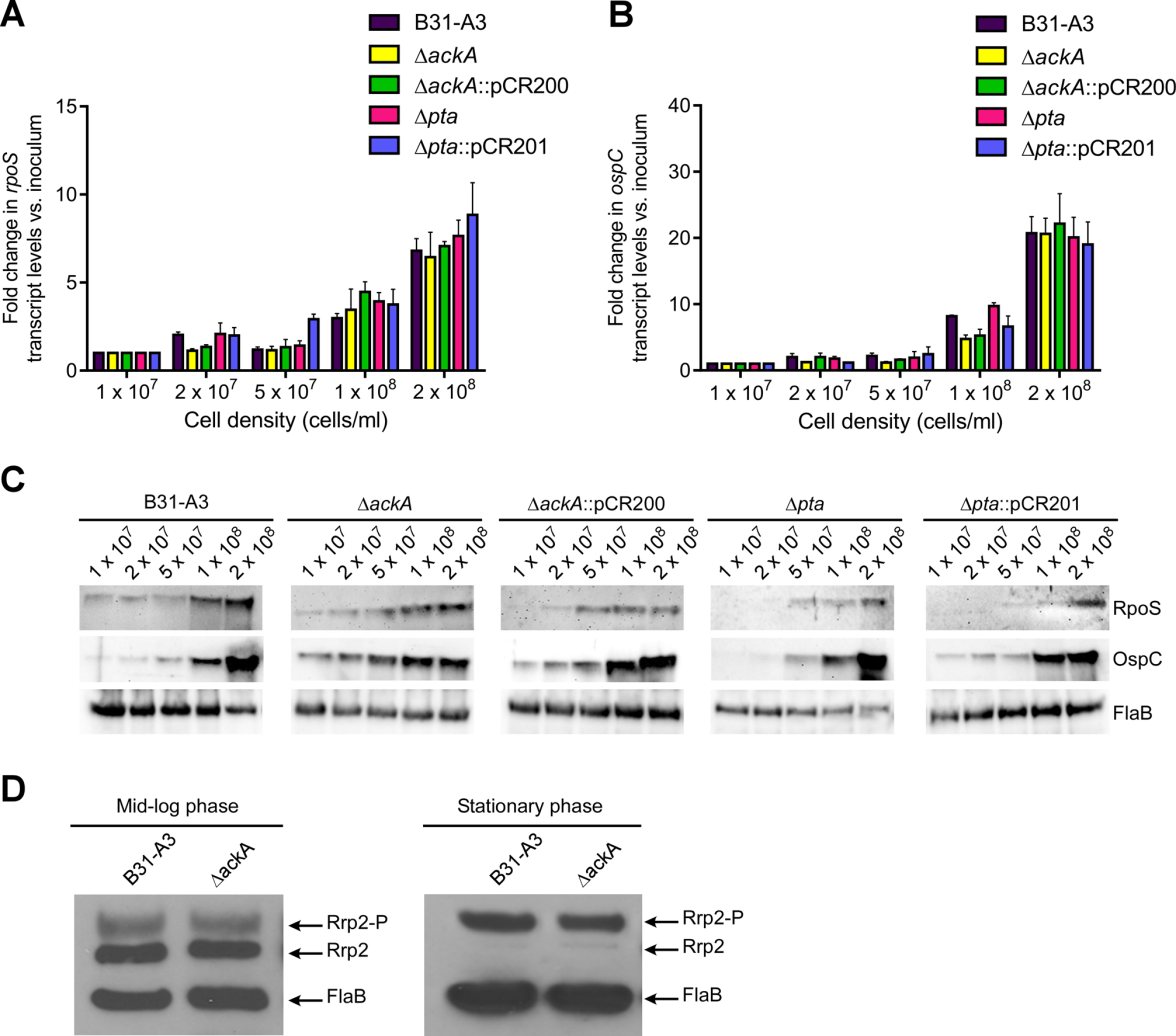
Effect of increasing cell density on *rpoS* and *ospC* transcript, RpoS and OspC protein expression, and levels of Rrp2 phosphorylation. (A) and (B) QRT-PCR analysis of *rpoS* and *ospC* transcripts in B31-A3, Δ*ackA*, Δ*pta*, Δ*ack*::pCR200 and Δ*pta*::pCR201 as each strain increased in cell density from 1 x 10^7^ to 2 x 10^8^ cells ml^-1^. (C) Immunoblot analysis of RpoS and OspC levels in each strain as cell density increased. Immunoblots were probed with antiserum specific for RpoS, OspC and FlaB. (D) Phos-Tag^®^ SDS-PAGE and immunoblot analysis of Rrp2 phosphorylation levels in B31-A3 and Δ*ackA* in mid-log and stationary phases of growth. Phosphorylated (Rrp2-P) and non-phosphorylated (Rrp2) proteins are indicated on the right. FlaB was included as protein load control for each assay.

It has been observed that as *B*. *burgdorferi* cells reach stationary phase/maximum cell density, the expression of RpoS and RpoS-regulated genes (e.g., *dbpA*, *ospC*) increases. To examine the role of the *ackA* and *pta* mutations on cell density-dependent regulation of *rpoS* and *ospC*, qRT-PCR was used to monitor transcript levels in B31-A3, Δ*ackA*, Δ*pta*, Δ*ackA*::pCR200, and Δ*pta*::pCR201 as the cell density increased. RNA was harvested from cell cultures at approximately 1 x 10^7^, 2 x 10^7^, 5 x 10^7^, 1 x 10^8^, and 2 x 10^8^ cells ml^-1^. Transcript levels were compared to the initial cell density (1 x 10^7^ cells ml^-1^) and normalized to *rpoD* transcripts. At low cell densities, 1–5 x 10^7^ cells ml^-1^, *rpoS* and *ospC* transcripts did not change significantly and no difference was observed between the strains (Fig 6A and 6B). As cells reached maximum cell density, 1–2 x 10^8^ cells ml^-1^, all strains showed a dramatic increase in *rpoS* and *ospC* transcripts. No significant difference was observed between the strains.

The levels of RpoS and OspC were analyzed by immunoblot in strains B31-A3, *ΔackA*, *Δpta*, *ΔackA*::pCR200, and *Δpta*::pCR201 at different cell densities (Fig 6C). RpoS levels were low at low cell densities and increased as cells reached 1 x 10^8^ cells ml^-1^ for each strain tested. OspC could also be detected at low levels at lower densities and increased concomitantly with an increase in *rpoS*, *ospC* and RpoS levels. Taken together, these results showed that the mutant strains, *ΔackA* and *Δpta*, are able to up-regulate *rpoS*, *ospC*, RpoS, and OspC, as cells reached stationary phase/maximum cell density as observed in the wild-type cells. These data suggest the AcP generated by AckA does not affect cell density-dependent regulation in *B*. *burgdorferi*.

The proposed phosphate donor for Rrp2 is AcP. Phosphorylation of Rrp2 activates RpoN, which in turn up-regulates *rpoS* and its regulon of virulence genes. qRT-PCR and immunoblot data suggested that deleting the gene (*ackA*) encoding the only known way to enzymatically synthesize AcP in *B*. *burgdorferi* had no effect on temperature- or cell density-dependent gene regulation. This suggested that in Δ*ackA*, the levels of phosphorylated Rrp2 (Rrp2-P) were unaffected by the loss of AckA and AcP. To test this hypothesis, cells were harvested at mid-log and stationary/maximum cell density phase of growth and assayed for the levels of Rrp2-P. As cells transitioned from mid-log to maximum cell density, the ratio of non-phoshorylated Rrp2 to Rrp2-P shifts dramatically in B31-A3 and Δ*ackA* (Fig 6D). More importantly, deleting *ackA* had no effect on the phosphorylation levels of Rrp2 at different stages of growth, confirming the qRT-PCR and immunoblot data. Taken together, these data strongly suggest that AcP does not play a significant role in cell density-dependent regulation through the Rrp2-RpoN-RpoS regulatory cascade.

### The absence of *ackA* did not change the effect of exogenous sodium acetate on RpoS and OspC levels

**Fig 7:**
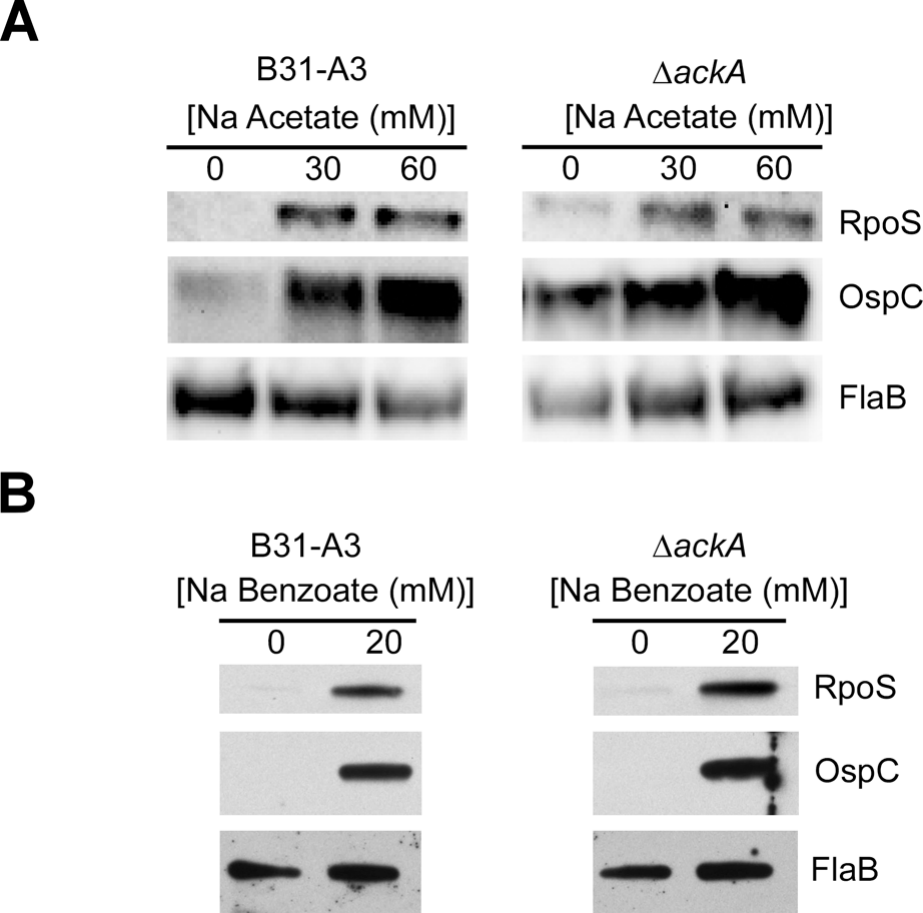
Effect of acetate on RpoS and OspC expression and Rrp2 phosphorylation. (A) Immunoblot analysis of RpoS and OspC in B31-A3 and Δ*ackA* in the presence of 0, 30, and 60 mM sodium acetate. (B) Immunoblot analysis of RpoS and OspC in B31-A3 and Δ*ackA* in the presence of 0 and 20 mM sodium benzoate. In each panel, FlaB was included as a protein load control.

Previous studies have shown that the addition of 20–90 mM sodium acetate at pH 7.0 leads to an increase in both *rpoS* and *ospC* transcript and RpoS and OspC protein levels in wild-type *B*. *burgdorferi* cells [11, 23]. In those studies, it was suggested that the addition of acetate stimulated the production of AcP via AckA, leading to an increase in Rrp2-P and activating the Rrp2-RpoN-RpoS regulatory cascade. To assess the effect of exogenous acetate on RpoS and OspC expression, B31-A3 and Δ*ackA* were exposed to increasing concentrations of sodium acetate (0, 30, and 60 mM) at pH 7.0. Both B31-A3 and Δ*ackA* showed increased RpoS and OspC expression when they were exposed to increasing concentrations of sodium acetate (Fig 7A), similar to previous reports. In addition, high concentrations of sodium acetate (30 and 60 mM) slowed the growth of both B31-A3 and Δ*ackA*, while lower concentrations did not affect growth (S1 Fig). Previously, in *E*.*coli*, it has been shown that weak permeant acids such as acetate or benzoate can trigger rapid changes in intracellular pH, leading to changes in gene regulation [24]. To assess whether sodium benzoate would elicit a similar response as sodium acetate in *B*. *burgdorferi*, B31-A3 and Δ*ackA* were incubated with 20 mM sodium benzoate. Similar to the expression observed with the addition of sodium acetate (Fig 7A), both strains showed an increase in RpoS and OspC levels when 20 mM sodium benzoate was added to the growth medium (Fig 7B).

### *ackA* and *pta* are required for infectivity in immunocompetent mice

To assess the infectivity of B31-A3, Δ*ackA*, and Δ*pta*, each strain was inoculated by intradermal injection into five RML mice (a closed colony of Swiss-Webster mice used at the RML since 1937) (3 separate replicates for a total of 15 mice injected per strain). B31-A3 was isolated from all of the mouse tissues tested (ankle joint, bladder, and skin). However, Δ*ackA* and Δ*pta* were non-infectious and were not isolated from any of the tissues tested. As previously shown, the Rrp2-RpoN-RpoS cascade appears to function normally in Δ*ackA* and Δ*pta* (Figs 5 and 6), so the inability of these strains to colonize mice is most likely due to other factors independent of Rrp2-RpoN- RpoS-dependent regulation. Because Δ*ackA* and Δ*pta* require mevalonolactone for survival *in vitro*, it is possible that the inability to colonize a mouse is due to very low levels of mevalonate in blood and tissue. We have previously shown that these mutants require a minimum of 100 μM mevalanolactone to survive *in vitro* (Fig 4). While the levels of mevalonate are difficult to assess in mice, experimental data has shown that the mevalonate concentrations in humans range from 28–43 nM, well below the levels required for the survival of Δ*ackA* and Δ*pta* [25], (http://www.hmdb.ca/metabolites/HMDB00227).The inability of these mutants to survive *in vivo* is most likely due to low levels of mevalonate in the host rather than a direct result of mutations in *ackA* or *pta* affecting gene regulation through the Rrp2-RpoN-RpoS regulatory cascade.

## Discussion

Acetate, AcP, and AcCoA are key metabolites in central metabolism, cell signaling and gene regulation in many eukaryotes, prokaryotes and some species of *Archaea* [16, 26, 27]. Most commonly, these molecules are produced during fermentation (acetogenesis). Typically, pyruvate dehydrogenase converts pyruvate to AcCoA and NAD^+^, with AcCoA being converted to AcP by phosphate acetyltransferase (Pta) and to acetate by acetate kinase (AckA) [16]. While *Borrelia* species have *ackA* and *pta* homologs, they do not appear to be using Pta and AckA for acetogenesis. There are several lines of evidence to support this conclusion. First, *B*. *burgdorferi* are homo-fermenters catabolizing glucose to lactate with no production of acetate [28, 29]. Second, they do not harbor a gene encoding pyruvate dehydrogenase. Third, they do not harbor genes encoding pyruvate oxidase or pyruvate formate lyase, which are enzymes capable of generating AcCoA from pyruvate. Finally, they harbor no genes encoding proteins for β-oxidation of fatty acids or a TCA cycle to generate and consume AcCoA for energy. Taken together, these data suggest a different role for *ackA* and *pta* in *B*. *burgdorferi*.

The utilization or activation of acetate in *Archaea* or *Bacteria* involves four possible pathways. In acetoclastic methanogens (e.g.,genera *Methnosarcina*), AckA, Pta and CO dehydrogenase/AcCoA synthase convert acetate to CO_2_ and CH_4_ [26]. Acetate uptake/utilization in Bacteria can be accomplished using: (i) irreversible AcCoA synthetase (AMP-forming) that generates AcCoA in a two-step reaction; (ii) irreversible AcCoA synthetase (ADP-forming) in a single step; or (iii) via the the Ack-Pta pathway. The *B*. *burgdorferi* genome does not contain genes annotated as AcCoA synthetase, indicating that acetate activation requires AckA and Pta. Due to the limited metabolic capabilities of *B*. *burgdorferi*, AcCoA, generated by these enzymes, appears to be used exclusively for the synthesis of mevalonate and activated isoprenoids. Ultimately, these isoprene units are condensed to C_55_-P for cell wall biogenesis (see Fig 1). The genome of *B*. *burgdorferi* harbors genes encoding enzymes for the complete synthesis of C_55_-P, lipid I, lipid II and peptidoglycan. While the enzymes required for the completion of the final steps of this pathway have not been characterized experimentally, evidence suggests that the downstream portion of the pathway is intact and functions as annotated. Lipid II molecules are susceptible to certain antibiotics, such as vancomycin, which binds and disrupts lipid II function, leading to cell death in bacteria containing this structure [20]. *B*. *burgdorferi* is sensitive to vancomycin and exposure to the drug disrupts cell integrity [19, 30]. Bacteria require significant amounts of lipid II for cell wall biosynthesis and growth, synthesizing up to 2 x 10^5^ copies of C_55_-P per cell [31, 32]. Using the pathway described above, *B*. *burgdorferi* uses an estimated 66 ATPs to synthesize one lipid II molecule representing a huge investment in energy to produce the required amount of this essential lipid [20, 31, 32].

The role of acetate in cell wall biogenesis in *B*. *burgdorferi* seemed to be quite clear. However, recent studies suggested a role for AcP in the Rrp2-RpoN-RpoS regulatory cascade [11, 23]. It had been proposed that low molecular weight phosphate donors, such as AcP, might play an important role in Rrp2-dependent regulation by directly phosphorylating Rrp2 independent of its cognate histidine kinase Hk2 [9, 11]. Previous experiments attempted to manipulate the intracellular concentration of AcP by adding sodium acetate to the growth media [11, 23]. In those studies, increasing concentrations of sodium acetate stimulated the synthesis of RpoS and OspC, suggesting that this is the case. However, it was not possible to measure the intracellular AcP concentration to confirm that exogenous acetate was responsible for the observed increase in RpoS and OspC.

AcP is a high energy form of phosphate and possesses a higher energy of hydrolysis than ATP [16]. Several studies have investigated the role of AcP in signal transduction via response regulator proteins [13, 15, 33]. In those reports, AcP acts as a high energy phosphate donor to response regulator proteins *in vitro*. However, under *in vivo* conditions, the contribution of AcP to response regulator activation is seen only when cognate histidine kinases are not present [i.e., null mutations and/or under very specific growth conditions] [16, 34]. Additionally, *E*.*coli* has multiple pathways that use or generate AcCoA and AcP, making it difficult to study the effects of AcP on global regulation [16]. This is not the case with *B*. *burgdorferi*. Due to its limited metabolic capabilities, AcP and AcCoA can only be derived from the linear pathway that synthesizes C_55_-P for cell wall assembly. To better understand the role of AcP in cell signaling in *B*. *burgdorferi*, *ackA* or *pta* was deleted in strain B31-A3. These mutants were selected by including mevalonolactone, an intermediate in the C_55_-P pathway, in the growth media. Growth experiments confirmed that Δ*ackA* and Δ*pta* had an absolute requirement for mevalonate and established that these genes were essential for the replication and viability of *B*. *burgdorferi*. More importantly, the strains allowed the evaluation of the putative role of AcP in mutants which are unable to synthesize AcP (Δ*ackA*) or could overproduce AcP (Δ*pta*) while maintaining a genetic background in which the cognate histidine kinase (Hk2) for Rrp2 was intact.

To analyze the role of AcP in cell signaling through the Rrp2-RpoN-RpoS cascade, experiments were conducted under conditions known to activate expression of the Rrp2-RpoN-RpoS-dependent genes. RpoS and its regulon has been shown to be activated in response to increases in temperature (23–34°C) and as cells reach maximum cell density (~2–3 X 10^8^ cells ml^-1^) *in vitro* [4, 10, 35]. These laboratory conditions are meant to mimic conditions the spirochete encounters as it transitions from its tick vector to a mammalian host. When B31-A3, Δ*ackA*, Δ*pta*, Δ*ackA*::pCR200, and Δ*pta*::pCR201 were exposed to a change in temperature or as the cells approached and reached maximum cell density, no significant differences were observed in the transcripts or the protein levels for *rpoS*, *ospC*, RpoS and OspC. In every case, the genes and proteins were activated similarly to wild-type cells, suggesting the AckA and Pta were not directly involved in these regulatory signals. However, there are several tiers of transcriptional, translational and post-translational regulation on *rpoS*, *ospC*, RpoS and OspC after Rrp2 is initially phosphorylated [7, 9, 36–38]. To complete these studies, we directly assayed the levels of Rrp2-P in the wild-type and Δ*ackA* strains as cells reached maximum cell density. No differences were observed in the ratio of Rrp2 and Rrp2-P in B31-A3 or Δ*ackA*, suggesting that AcP was not required for phosphorylation of Rrp2. Taken together, these data strongly suggest that AckA and Pta, or the products of their enzymatic activity (AcP and AcCoA), do not appear to be required to modulate the levels of Rrp2-P or to activate the Rrp2-RpoN-RpoS cascade under the conditions tested.

Xu et al. used high concentrations of acetate in *B*. *burgdorferi* to attempt to influence the intracellular concentration of AcP and assess its potential as a small molecular weight phosphate donor for Rrp2 [11]. When high concentrations of acetate were added to the growth medium, an increase in RpoS and OspC was observed and attributed to increased levels of cytoplasmic AcP. However, when strains B31-A3 and Δ*ackA* were exposed to high concentrations (30 and 60 mM) of sodium acetate, production of RpoS and OspC were the same in both strains. These data raised a fascinating question. If high concentrations of sodium acetate increased the production of RpoS and OspC independent from the intracellular concentration of AcP, what was the effect being observed? Wilks and Slonczewski, using GFP and YFP gene fusions, were able to show that the addition of high concentrations of sodium acetate (20mM) to these *E*. *coli* reporter strains triggered a very rapid fall in intracellular pH (7.5–5.5) [24]. Sodium benzoate elicited a similar response in the *E*.*coli* strains tested, showing that the intracellular pH did not recover from exposure to permeant organic acids. When B31-A3 and Δ*ackA* cells were exposed to 20 mM sodium benzoate, an increase in the synthesis of RpoS and OspC was observed in both strains, similar to the increase that was observed with the addition of sodium acetate. Therefore, we believe that the addition of sodium acetate to *B*. *burgdorferi* is not triggering increased levels of RpoS and OspC via AcP, but instead that weak permeant acids are initiating a pH/acid stress response modulated by increased levels of RpoS. Currently, we are investigating the mechanism responsible for the regulation of the acid stress response in *B*. *burgdorferi*.

## Experimental Procedures

### Bacterial Strains, Culture Conditions and Reagents

*E*. *coli* was used for cloning and plasmid maintenance. *B*. *burgdorferi* strain B31-A3 [39] and derivatives were grown in Barbour-Stoenner-Kelly medium (BSKII) at pH7.6 or pH6.8 at 34°C unless stated otherwise. Spirochetes were enumerated by dark-field microscopy. Antibiotics were used at the following concentrations: chloramphenicol and gentamicin at 20 μg ml^-1^, streptomycin at 50 μg ml^-1^, and spectinomycin at 100 μg ml^-1^. All chemicals were purchased from Sigma-Aldrich (St Louis, MO) unless stated otherwise.

### Generation of B31-A3Δ*ackA* (*bb0622*) and B31-A3Δ*pta* (*bb0589*) Mutants and Complementing Vectors

Approximately 0.6 kb of DNA 5’ and 3’ of *ackA* (*bb0622*) or *pta* (*bb0589*) open reading frames were amplified by PCR using primers 1 & 2 and 3 & 4 for *ackA* and 9 & 10 and 11 & 12 for *pta* (Table 1). The *aacC1* and *aadA* genes, including the *flgB* promoter, were amplified by PCR from pBSV2G or pKFSS1 respectively, using primers 17 & 18 or 19 & 20 (Table 1). To generate pCR100 (pPCR-Script Cam::Δ*ackA*::*aacC1*) and pCR101 (pPCR-Script Cam::Δ*pta*::*aadA*) for allelic exchange, the upstream region, antibiotic resistance cassette (*flgB*p-*aacC1* for Δ*ackA* or *flgB*p-*aadA* for Δ*pta*) and downstream region of each targeted gene were sequentially cloned into pPCR-Script Cam SK(+) (Agilent Technologies, Cedar Creek, TX) using appropriate restriction enzymes. Relevant plasmid sequences were confirmed by sequencing (ACGT Inc., Wheeling, IL). Wild type *B*. *burgdorferi* was transformed with pCR100 or pCR101 as previously described [39] and transformants were selected by plating in BSKII medium supplemented with 10 mM mevalonolactone and the appropriate antibiotic. Antibiotic resistance cassette insertion sites were confirmed by PCR using primers 7 & 8 (Δ*ackA*) or 15 & 16 (Δ*pta*) (Table 1).

The B31-A3Δ*ackA* and B31-A3Δ*pta* strains, subsequently referred to as Δ*ackA* and Δ*pta* respectively, were complemented using pCR200 (pKFSS1::*ackA*) or pCR201 (pBSV2G::*pta*) respectively. Briefly, *ackA* and 600 bp of DNA 5’ of the coding region, or the *pta* gene and 423 bp of DNA 5’ to the coding region were amplified by PCR using primers 5 & 6 or 13 & 14 (Table 1) and cloned into appropriately digested pKFSS1 [41] or pBSV2G [40], respectively. The resulting plasmids, pCR200 or pCR201, were transformed into Δ*ackA* or Δ*pta* as previously described and transformants were plated in BSKII medium (lacking mevalonolactone) and containing the appropriate antibiotics. PCR and sequencing (ACGT Inc.) was used to confirm both strains.

### Mevalonolactone Dependence, Temperature Shift, Cell Density and Acid Stress Experiments

B31-A3, Δ*ackA*, Δ*pta*, Δ*ackA*::pCR200 and Δ*pta*::pCR201 were analyzed for their ability to survive and replicate in BSKII media with or without mevalonolactone as well as to determine the minimum mevalonolactone concentration required for growth. Cultures were grown in 50 ml BSKII (+10 mM mevalonolactone) to mid-logarithmic phase and cells were washed three times with HN buffer (20 mM HEPES, pH 7.6, 50 mM NaCl) to remove residual mevalonolactone. Equivalent spirochete numbers were inoculated into BSKII media containing varying concentrations of mevalonolactone. Growth and survival was determined using viable cell counts, assayed by plating in BSKII (+10mM mevalonolactone) solid media.

Temperature shift experiments were performed as previously described with minor modifications [42, 43]. Briefly, *B*. *burgdorferi* strains were grown in 10 ml of BSK II (pH 6.8) at 23°C under a microaerobic atmosphere (90% N_2_, 5% CO_2,_ 5% O_2_) to mid-log phase and expanded to 500 ml at 1 x 10^6^ cells ml^-1^. Cultures were grown to 1 x 10^7^ cells ml^-1^ and then shifted to 34°C. Samples were harvested for RNA and protein analysis at 0, 4, 8, 24, and 48 h time points following the temperature shift. The pH of the culture medium after 48 h of incubation was 6.8.

For cell density experiments, *B*. *burgdorferi* cultures were grown in 10 ml of BSK II medium at pH 6.8 under a microaerobic atmosphere to mid log phase and subsequently expanded to 500 ml at 1 x 10^6^ cells ml^-1^. Aliquots were harvested for RNA and protein analysis at cell densities of 1 x 10^7^ cells ml^-1^, 2 x 10^7^ cells ml^-1^, 5 x 10^7^ cells ml^-1^, 1 x 10^8^ cells ml^-1^, and 2 x 10^8^ cells ml^-1^. Two 10 ml samples were harvested at each time point or cell density for RNA and protein analysis.

Growth of *B*. *burgdorferi* in increasing concentrations of extracellular sodium acetate or sodium benzoate was performed as previously described [11]. Briefly, *B*. *burgdorferi* B31-A3 and Δ*ackA* were grown from frozen stock in BSK II containing 10 mM mevalonolactone at pH 6.8 under microaerobic conditions. Cells were subcultured to a density of approximately 2 x 10^6^ cells ml^-1^ in BSK II containing 10 mM mevalonolactone and either increasing concentrations of sodium acetate (0, 30, and 60 mM) or sodium benzoate (0 and 20 mM). B31-A3 and Δ*ackA* cells were incubated with acetate or benzoate until cell density reached approximately 2 x 10^7^ cells ml^-1^ at which point each culture was harvested for western blot analysis (described below).

### RNA Extraction and Quantitative Reverse Transcription Polymerase Chain Reaction (qRT-PCR)

Samples for RNA analysis were harvested at 4°C by centrifugation, the cell pellet resuspended in Trizol solution and the RNA extracted according to the manufacturer’s instructions (Invitrogen, Grand Island, NY). RNA samples were subsequently treated with TURBO DNase (Ambion, Austin, TX), following the manufacturer’s instructions. Quantitative RT-PCR was performed using a one-step QuantiTect SYBR Green kit (Qiagen) following the manufacturer’s instructions. To confirm gene deletions, *ackA* and *pta* transcripts were assayed in WT, Δ*ackA*, Δ*pta* and their complements using primers 21–24 (Table 1). To amplify *rpoD*, *ospC* and *rpoS* transcripts, primers 25–30 were used (Table 1). cDNA was synthesized at 42°C for 30 minutes and denatured at 95°C for 15 minutes. Quantitative RT-PCR was performed under standard conditions on a LightCycler (Roche, Indianapolis, IN). The LightCycler software was used for the analysis of fold changes and *rpoD* normalization.

### SDS-PAGE Gel Electrophoresis, Immunoblot and Phos-tag analysis

Protein samples were prepared by harvesting cells by centrifugation (8,000 x g, 10 min, 4^○^C), washing the cell pellet with HN buffer and lysis in B-per lysis solution (Merck KGaA, Darmstadt, Germany). Cell debris was removed by centrifugation and the supernatant was collected. Protein concentration was quantified by absorbance and equivalent amounts were loaded onto 4–20% Tris-HEPES polyacrylamide gels (NuSep, Bogart, GA). Proteins were transferred to nitrocellulose membranes (Invitrogen) and immunoblot analysis was performed as previously described (Burtnick et al. 2007). Proteins were visualized with the ECL^TM^ Plus Western Blotting Detection Reagents (GE Healthcare, Pittsburgh, PA). Primary antibodies were used at the following concentrations: α-RpoS polyclonal antiserum (1:200), α-OspC polyclonal antiserum (1:1000), α -flagellin monoclonal antibody (H9724 1:50) [44], Rrp2 polyclonal antiserum (1:200) [22].

Rrp2 phosphorylation was assessed using Phos-tag analysis [45]. Bacterial cells grown to mid-log and stationary phase as described above and were harvested by centrifugation. The cell pellets were resuspended in SDS-PAGE loading buffer and loaded onto gels containing 75 μM Phos-Tag^TM^ acrylamide and 150 μM Zn(NO_3_)_2_. Following electrophoresis, the gels were washed 15 min in transfer buffer (25 mM Tris, 192 mM glycine, 1mM EDTA), and 15 min in transfer buffer without EDTA before transferring to a nitrocellulose membrane.

### Promoter Analysis of *ackA* and *pta* using 5’-primer Extension Analyses

Primer extension analysis was performed using the 5’ RACE kit (Invitrogen) according to the manufacturer’s instructions. Briefly, cDNA synthesis was performed using gene specific primers (GSP) listed in Table 1. cDNA was purified and used in TdT reactions that added a poly-cytosine tail to the 5’ end of the cDNA. Tailed cDNA was subsequently used in PCR reactions with an internal GSP primer (*ackA*-GSP2 or *pta*-GSP2—Table 1) and an abridged anchor primer provided by Invitrogen. PCR products were analyzed on an agarose gel, purified on a Qiaquick PCR purification column (Qiagen) and subjected to Sanger sequencing (ACGT Inc.).

### Analysis of Infectivity of Δ*ackA* and Δ*pta* in a Murine Model

*B*. *burgdorferi* B31-A3 and mutant strains were grown to mid log phase and three groups of five female RML mice were obtained from the Rocky Mountain Laboratories Veterinary Branch (RMVB) breeding facility. Each mouse was inoculated by intradermal injection with an inoculum of 5 x 10^5^ cells grown in BSKII. The mice used in this study were monitored regularly with health checks occurring twice daily. Inocula were plated to confirm dose and plasmid content. Three weeks post-inoculation, ear punch biopsies were performed. Seven to 14 days following initial screening, mice were anesthetized by inhalation of isoflurane and, while under anesthesia, were euthanized humanely by cervical dislocation. Tissues from the ear, bladder, liver, and ankle joint tissues were cultured in BSK II containing 10 mM mevalonolactone to test for spirochete positive tissue. Mouse infection studies were carried out in accordance with guidelines of the National Institutes of Health. All animal work was done according to protocols approved by the Rocky Mountain Laboratories Animal Care and Use Committee (Protocol Number 2014–021).

## Supporting Information

S1 Fig Effect of acetate on growth of *B*. *burgdorferi*. (A) Growth of B31-A3 and (B) Δ*ackA*, respectively in the presence of 0, 30, and 60 mM exogenously added sodium acetate.
(TIF)
