# Supplementary figures and images for: Acetyl-Phosphate Is Not a Global Regulatory Bridge between Virulence and Central Metabolism in Borrelia burgdorferi
Source: PLoS One. 2015 Dec 17;10(12):e0144472. doi: 10.1371/journal.pone.0144472 (PMC4683003; doi:10.1371/journal.pone.0144472)

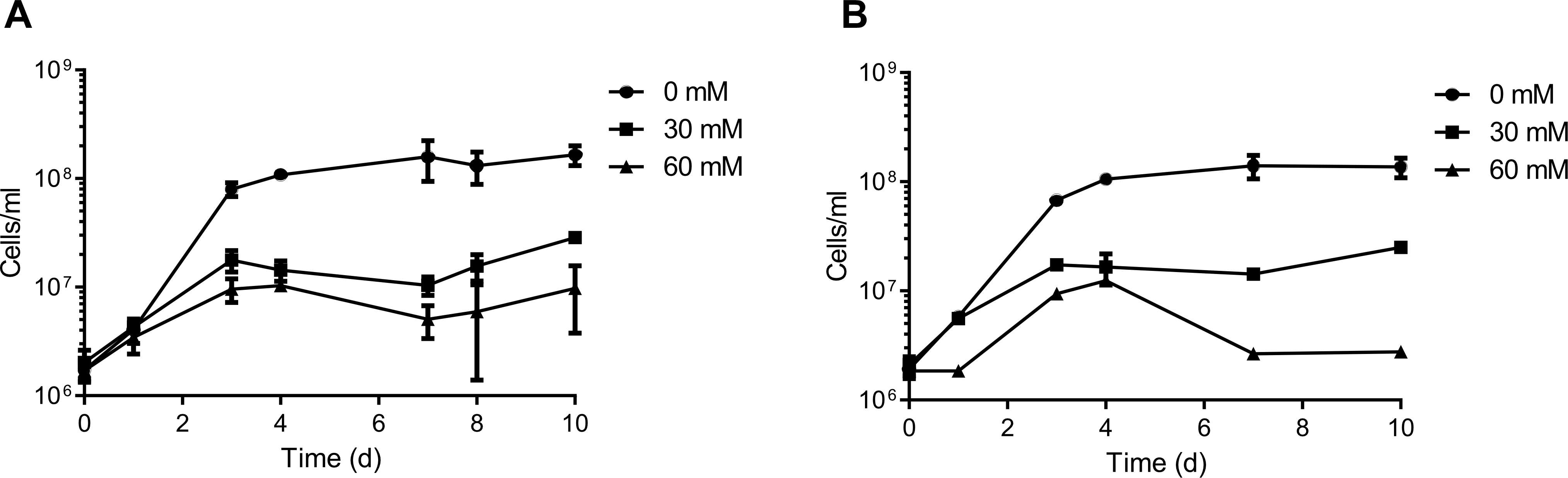

Supplement: S1 Fig — (A) Growth of B31-A3 and (B) ΔackA, respectively in the presence of 0, 30, and 60 mM exogenously added sodium acetate. (TIF) [file pone.0144472.s001.tif]
